# Three-Dimensional Adaptive Image Compression Concept for Medical Imaging: Application to Computed Tomography Angiography for Peripheral Arteries

**DOI:** 10.3390/jcdd9050137

**Published:** 2022-04-27

**Authors:** Guillaume Fahrni, David C. Rotzinger, Chiaki Nakajo, Jamshid Dehmeshki, Salah Dine Qanadli

**Affiliations:** 1 Cardiothoracic and Vascular Division, Department of Diagnostic and Interventional Radiology, Lausanne University Hospital and University of Lausanne, 1011 Lausanne, Switzerland; david.rotzinger@chuv.ch (D.C.R.); cnakajo@cliniquevalere.ch (C.N.); salah.qanadli@chuv.ch (S.D.Q.); 2 Imaging and Image-Guided Therapies Lab (IGT-L), University of Lausanne, 1015 Lausanne, Switzerland; 3 Department of Computer Science, Kingston University, Kingston-upon-Thames KT1 2QT, UK; j.dehmeshki@kingston.ac.uk

**Keywords:** medical image compression, computer aided segmentation, image processing, radiology, peripheral artery disease, CT angiography

## Abstract

Advances in computed tomography (CT) have resulted in a substantial increase in the size of datasets. We built a new concept of medical image compression that provides the best compromise between compression rate and image quality. The method is based on multiple contexts and regions-of-interest (ROI) defined according to the degree of clinical interest. High priority areas (primary ROIs) are assigned a lossless compression. Other areas (secondary ROIs and background) are compressed with moderate or heavy losses. The method is applied to a whole dataset of CT angiography (CTA) of the lower extremity vasculature. It is compared to standard lossy compression techniques in terms of quantitative and qualitative image quality. It is also compared to standard lossless compression techniques in terms of image size reduction and compression ratio. The proposed compression method met quantitative criteria for high-quality encoding. It obtained the highest qualitative image quality rating score, with a statistically significant difference compared to other methods. The average compressed image size was up to 61% lower compared to standard compression techniques, with a 9:1 compression ratio compared with original non-compressed images. Our new adaptive 3D compression method for CT images can save data storage space while preserving clinically relevant information.

## 1. Introduction

Datasets generated from medical imaging applications reach a volume that does not allow easy archiving or transmission by Picture Archiving and Communications Systems (PACS) [1,2]. This volume is even expected to increase significantly in the coming years with continuing technological improvements [3,4]. Compression technologies have been applied to reduce the volume of medical images for storage and transmission purposes across networks with various bandwidths [5,6,7,8]. Several lossy and lossless compression methods have been proposed in the literature [9,10,11,12,13,14,15,16,17,18,19,20]. For lossy methods, some information is lost as a high compression ratio is the primary goal. Conversely, the exact original image can be recovered from the compressed image with lossless methods, but the compression rate is more modest. In general, lossy methods are good candidates to be applied to large volume medical imaging datasets. However, this might lead to a loss of important clinical information, and thus such methods cannot be applied blindly to medical datasets [21,22]. A hybrid method is needed to take advantage of lossy methods and preserve clinically meaningful information. One such hybrid approach is employed in JPEG2000 (JP2K) [23,24]. This compression technology introduces a region of interest (ROI) feature to avoid the loss of information in defined parts of an image that are more important than others. Typically, ROIs and the compression rate are determined manually, requiring substantial user interaction.

In the current clinical setting, lossless compression methods are widely preferred, due to fear of losing important clinical information with lossy methods, despite the significant compression gain they provide. There is a need for a better hybrid method that tackles the challenge of being usable in a clinical setting. Such a method needs to emphasize keeping the important clinical features and image quality while still providing a better compression rate than lossless methods.

We thus developed a new technique, based on the concept of an ROI-based approach to compression of image datasets. The new method integrates multiple ROIs, defined according to their content (adaptive system), and is applied to the entire dataset (3D approach). Our approach differs from other hybrid methods in the way that it includes multiple ROIs with different levels of compression depending on the clinical interest, with a primary ROI closely adapted to the clinically important part of the images, leading to a clinically driven compression method. The concept is applied to high-resolution CT angiography (CTA) of the lower extremities, which is routinely used to assess peripheral arterial diseases. In the proposed method, the contextual regions of interest correspond to a higher degree of clinical interest, i.e., the lower limb arterial tree.

Apart from reporting the new concept, this study aims to compare the quantitative/qualitative image quality and the file size reduction capability achieved by the new method with existing compression technologies.

## 2. Materials and Methods

Ten CTA datasets of the lower extremities were collected from the University Hospital of Lausanne, Switzerland. We included exams with arterial injection time and excluded patients with poor injection, completely occluded vessels or missing limbs. Images were stored in DICOM format, with a 512 × 512 voxel in-plane resolution, a bit depth of 16 bpp, a physical pixel dimensions 0.7 mm × 0.7 mm, and a slice thickness of 1.25 mm. X-ray tube current varied from 134 to 381 mA and the voltage was 120 kVp. Each CTA contained 1000 images. An in-house computer-aided segmentation (CAS) algorithm implemented in MATLAB was applied to detect the lower limb arteries automatically. A graphic user interface was developed for visually assessing the compressed images.

**Figure 1:**
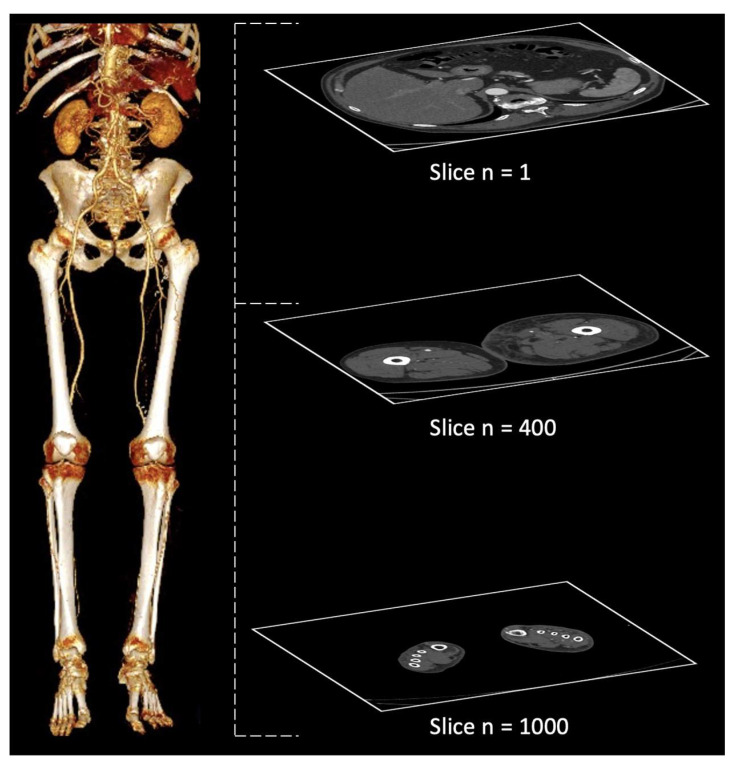
Example of a full 3D CTA scan of the lower limbs from slice 1 (proximal) to slice 1000 (distal): on the left, reconstructed volume-rendered images displaying the arterial tree; on the right, single slices at different levels.

An adaptive and ROI-based approach to image compression is proposed. A typical CTA of the lower limbs requires more than 1000 high-resolution images (Figure 1). However, the arteries, which are the target region for physicians, take up less than 5% of the image. Hence, developing an adaptive ROI-based 3D image compression method can fulfill a valuable clinical function.

**Figure 2:**
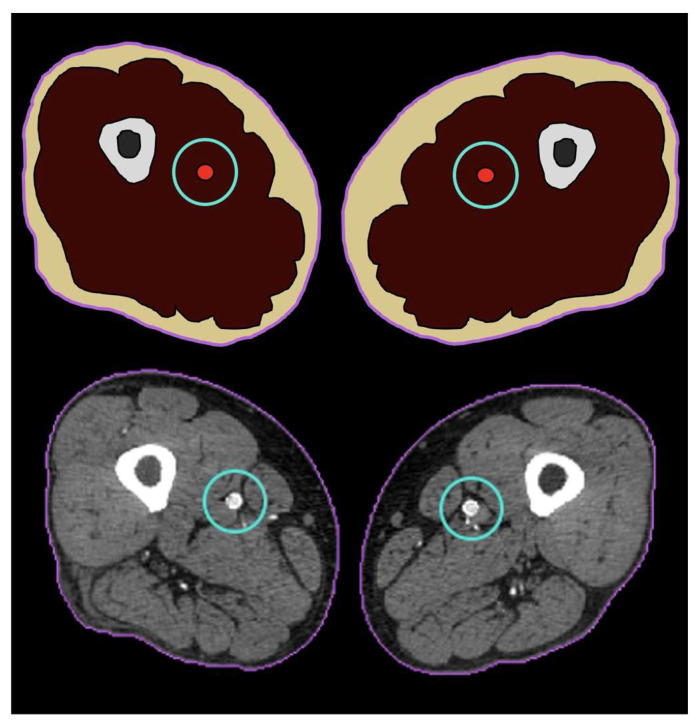
The three defined ROIs, schematic (**up**) and slice example (**down**): the PROI (blue circle) is centered around the arteries; the SROI (purple circle) defines the body, excluding the PROI; and the background includes the rest of the image.

We propose to split the images into three regions: primary region of interest (PROI), secondary region of interest (SROI), and background (Figure 2).

The PROI is the main region of interest in an image. The radiologist can define this region manually or automatically by a CAS [25,26]. The PROI contains the most critical information, and, consequently, this region should be preserved. It will undergo lossless compression, leading to minimal size reduction but ensuring the most crucial information of the exam is fully preserved.

In our application, the arterial tree is defined as the PROI. Our CAS performs automated segmentation of the arterial tree, which is then enlarged to the empirical value of eight times the artery surface to minimize the chance of missing part of the arteries due to under-segmentation. The resulting PROI region identifies the most clinically relevant part of the image.

The SROI contains regions of lesser clinical interest that can be compressed with moderate loss while keeping good visual quality. In our scenario, it is defined as the remaining body tissue apart from the PROI, representing approximately 50% of the image. As for the PROI, the SROI is automatically segmented by the CAS. Lossy compression is used to compress the SROI using medium compression rates to ensure all diagnostically relevant information is preserved with sufficient detail. The algorithm can operate either with a constant or a variable bit rate (detailed below) to compress the SROI data [27].

The background includes the rest of the image, which has no significant value. It corresponds to the area outside the human body and is compressed with a very high compression rate.

First, we evaluated if the tested algorithms provided images of sufficient quality, both in terms of quantitative and qualitative criteria. We compared the performances of three methods: (1) the proposed method with variable bit rate (MVAR), (2) the proposed method with fixed bit rate (MFIX), and (3) a standard lossy JPEG2000 compression without ROI. In the MVAR version, the compression bit rate is calculated based on the peak signal-to-noise ratio (PSNR) between consecutive slices, with a 40 dB minimum threshold. In the MFIX version, the compression bit rate of each slice is estimated as a mean of the compression bit rates obtained from the first version. For the lossy JPEG2000 without ROI method, the compression bit rate is the same as with MFIX. Lossless compression techniques were not included in this image quality evaluation since they only provide size reduction on compression and do not alter the original image.

The quantitative image quality was evaluated with the PSNR and Mean-Squared Errors (MSE) parameters, commonly used to assess the degree of distortion in compressed images [28,29]. In this algorithm, the minimum PSNR requirement for a high-quality encoding is assumed to be 40 dB. Above this threshold, original and reconstructed images are virtually indistinguishable by human eye [30].

The qualitative image was evaluated with a Mean Opinion Score (MOS) scale [31], ranging from 1 to 5 (Table 1). Although its usefulness can be a matter of debate [32], the MOS scale offers an alternative way to measure quality and represent how humans assess it. Two qualified radiologists evaluated the quality of the compressed images without consulting each other, in a blinded fashion.

A statistical non-parametric Kruskal–Wallis test and a post hoc pairwise Dunn test were applied to the MOS scores of the different tested methods. *p*-values were corrected using the Holm method. Statistical significance was set at *p* ≤ 0.05.

Secondarily, we assessed image size reduction. We compared the performance of four different methods: (1) MVAR, (2) lossless JP3D, (3) lossless JPEG2000, and (4) lossless H.264 method [33,34,35]. The JP3D VM version 1.1 and H.264 joint-reference model (JM) version 18.2 were used for the evaluation test. The four methods were compared with the non-compressed images in terms of image size reduction and compression ratio (CR). Because the MFIX and lossy JP2D method did not provide sufficient qualitative image quality (see Section 3.1), they were excluded from the file size reduction analysis.

## 3. Results

### 3.1. Image Quality

**Figure 3:**
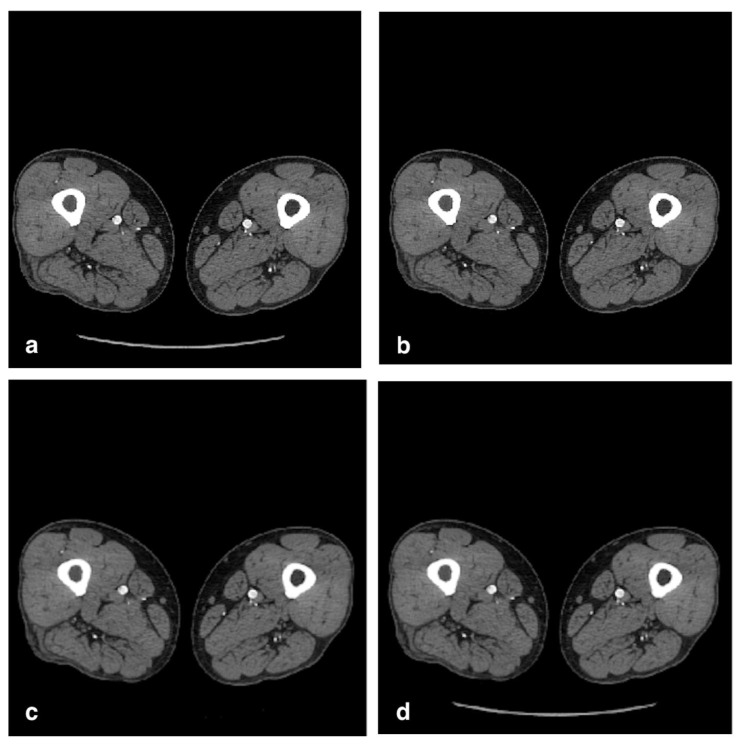
Image quality comparison with three different compression methods: (**a**) original image, slice 500, 512 × 512 (DICOM-16bit); (**b**) proposed method with variable bit rate (MVAR); (**c**) proposed method with fixed bit rate (MFIX); (**d**) lossy JPEG2000 without ROI image.

CTA images resulting from the three compression methods (MVAR, MFIX, and lossy JPEG2000 without ROI) are displayed in Figure 3.

**Figure 4:**
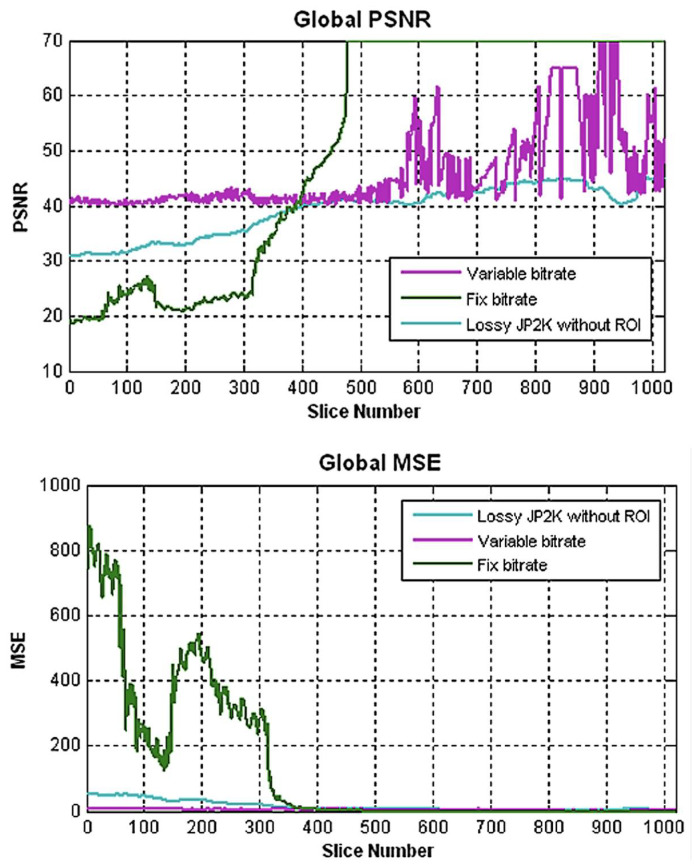
Comparison of the peak signal-to-noise ratios (PSNR) and mean-squared errors (MSE) between the three different evaluated compression methods for a single 1000-slice CTA from the sample dataset.

Quantitative image quality metrics (PSNR and MSE) are presented in Table 2 and plotted in Figure 4. PSNR remained above the 40 dB threshold only for the MVAR method, while it fell well below 40 dB on proximal CTA slices in the two other methods. Proximal CTA slices are noisier due to the higher amount of soft tissue in the abdomen and pelvis, thus reducing the PSNR. MSE followed the same trend, with high values for the first 300 slices.

The average slice size and MSE for the three methods are reported in Table 3. The average slice size was lower with the MFIX method, while the MSE was lower with the MVAR method.

**Figure 5:**
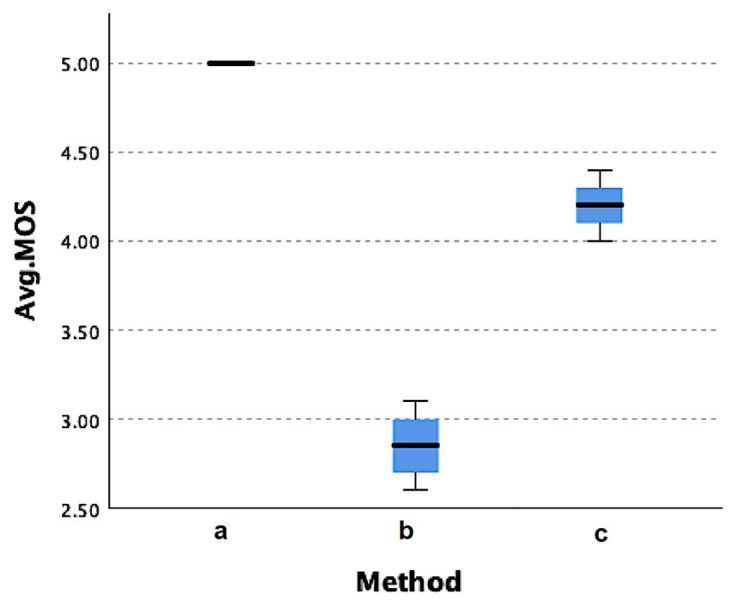
Boxplot of the MOS scores for the three tested methods. (a) Proposed method with variable bit rate (MVAR); (b) Proposed method with fixed bit rate (MFIX); (c) Lossy JPEG2000 without ROI.

The diagnostic MOS scale based on the review of two radiologists is shown in Table 4 and Figure 5. Average MOS was good or better for the MVAR and lossy JPEG2000 scenarios. For the MFIX scenario, lower ratings were attributed to slice numbers beyond 400, i.e., in the distal anatomic region below the knee level.

The Kruskal–Wallis test showed statistically significant (*p* < 0.001) differences for the distribution of MOS scores across the three tested methods. The post hoc Dunn test showed that all three methods were statistically different (*p* < 0.05) from each other, with the most significant difference between the MVAR and the MFIX methods (*p* < 0.001).

### 3.2. Image Size Reduction

The four compared methods for image size reduction evaluation (MVAR, lossless JP3D, lossless JPEG2000, and lossless H.264) are presented in Table 5, with a comparison in terms of the average size of the DICOM slices and compression ratio (CR) for the 10 datasets. The MVAR method provided the best results compared to the other methods, with compression ratios ranging from 3:1 to 3.6:1 for the JPEG2000, JP3D, and H.264 scenarios, while being as high as 9:1 for the MVAR scenario.

## 4. Discussion

The MVAR method provided the best results for all evaluated compression methods. The MSE values for the MVAR method were much lower than for the two other methods for slice numbers less than 400. Regarding the PSNR values, only the MVAR method obtained values above the 40 dB threshold for the whole dataset. This is an important result, as it indicates that only the MVAR method can produce images with suitable quantitative quality parameters. The sudden transition in image quality around slice number 400 is thought to be due to the change in the density of body tissue between the proximal and the distal slices. The variable-bit-rate algorithm is designed to not fall under the 40 dB threshold, which is evaluated continuously, slice by slice. The result is a serrated PSNR variation in slices 600 to 1000, as the algorithm aims to increasingly compress after each slice but has to drop back when nearing the threshold.

Qualitative image evaluation results were in agreement with quantitative analysis metrics. The MVAR method provided the best results with MOS scores averaging 5. MOS scores also confirmed that the MFIX method provided images of insufficient quality for the slices lower than 400. As for the JPEG2000 without ROI method, MFIX provided intermediate results.

In terms of file size evaluation, the MVAR method average compression ratio was considerably higher compared with the other techniques, with a file size reduced from 512 to 56 kB, resulting in a high compression ratio of 9:1. This is a significant improvement compared to the low to medium compression ratios of lossless methods, while respecting the lossless constraint in the PROI and keeping an acceptable appearance in the SROI. In other words, the average size of the original images can be reduced up to 89% with respect to the original image, and up to 61% with respect to the lossless JPEG2000 standard, with no clinical impairment in image quality and diagnosis.

Segmentation of the peripheral arteries of the lower limb with our algorithm provided good delineation of the PROI in this investigation. CAS has the advantage of being fully automatic and highly reproducible. This is a necessity, as any other segmentation technique requiring even minimal manual inputs or interactions would be very challenging to implement in clinical practice, if not impossible. With further development, the segmentation algorithm can be implemented to artery segmentation of other parts of the body, with potential application to different CTA types. The aim of PROI detection is not to accurately segment the peripheral arteries. Therefore, the ROI segmentation method has been developed very conservatively to ensure the peripheral artery has been fully extracted. This may result in over-segmentation of the arterial tree, which may contain other tissues, such as bone. However, as PROI is only a small percentage of the total image, this has a negligible impact on the overall compression rate. Without this precaution, the algorithm could be at risk of under-segmenting the arterial tree, resulting in potentially clinically impactful loss of information in the PROI, which is to be avoided at all costs.

Segmentations of the SROI and background are also easy to obtain. This process is not expected to fail since the difference between the patient and the surrounding background is visually and mathematically significant. Nonetheless, it is essential to make sure that this segmentation is robust since any segmentation mistake could result in labeling parts of the patient as background, resulting in full compression of the region and providing clinically non-interpretable images.

Another advantage of our proposed method is that it is independent of the standard compression engine. In other words, the technique could be used in any ROI-based compression technique. In the future, with the potential emergence of new compression or new segmentation techniques, especially with the ongoing development of artificial intelligence (AI) based methods [36,37], the concept of a multi-level compression approach could still be used, with automatic segmentation of any pre-defined PROIs, SROIs, and background, to achieve even greater results in terms of compression ratio, while maintaining satisfactory image quality. Although the reduced image size in our proposed approach finds its main application in data storage optimization (i.e., image archiving), it could also benefit other domains in radiology, for example in the growing field of teleradiology [38,39], where it could lead to faster data transfers and reduced bandwidth requirements.

We believe that the proposed method adds an important contribution to the field of medical image compression techniques, as its hybrid nature fills a gap between lossless compression techniques that are currently used in clinical practice but provide low compression rates, and lossy compression techniques that provide good compression rates but are not usable in a clinical setting due to unreliable quality. The clinically driven multiple ROI approach is widely adaptable to different clinical scenarios. In addition, its automated nature makes it realistically usable in a clinical routine.

This study is subject to several limitations. Firstly, the PROI was enlarged to a value of eight times the artery surface to make sure all clinically relevant information was preserved. However, this value is empirical and could be further optimized and adjusted to any other criterion, depending on the clinical needs. Secondly, segmentation of the ROIs was homemade; nevertheless, our proposed concept can be applied using any other type of segmentation algorithm. Lastly, we did not evaluate the compressed datasets in a clinical setting to assess any change of diagnostic performance compared with non-compressed images.

## 5. Conclusions

We proposed an innovative adaptive approach to compress 3D digital medical images that can keep all necessary information, with lossless preservation of a defined PROI. Only some diagnostically irrelevant information may be lost in the compression process, with no impact on clinical diagnosis for the SROI. Interaction with the user is minimized by the automatic segmentation of ROIs and the automatic compression algorithm. The size of the resulting images was significantly reduced by as much as 61% when compared with standard lossless algorithms (JPEG2000, JP3D, and H.264), with compression rates as high as 9:1 with respect to the original images, whilst maintaining the diagnostic quality. Our proposed method was applied on peripheral artery CT angiography images, but could be extended to other body parts and other medical image applications.

## Figures and Tables

**Table 1:** Mean Opinion Score (MOS) for the qualitative image analysis.

Score	Quality	Impairment
5	Excellent	Imperceptible
4	Good	Perceptible but not annoying
3	Fair	Slightly annoying
2	Poor	Annoying
1	Bad	Very annoying

**Table 2:** Quantitative measurements in terms of PSNR (in dB) and MSE for different CTA slices: (MVAR) proposed method with variable bit rate; (MFIX) proposed method with fixed bit rate; and (JP2K) lossy Jpeg2000 without ROI.

Slice No.	PSNR_MVAR_	PSNR_MFIX_	PSNR_JP2K_	MSE_MVAR_	MSE_MFIX_	MSE_JP2K_
1	40.70	19.31	31.00	5.52	761.83	51.59
100	40.25	23.96	31.43	6.13	260.86	46.71
200	41.78	21.43	32.92	4.31	466.91	33.14
300	42.10	23.46	35.47	4.00	292.95	18.44
400	40.46	42.51	39.96	5.83	3.64	6.55
500	40.98	78	41.28	5.18	0.001	4.83
600	54.02	78	40.38	0.25	0.001	5.95
700	43.29	78	42.43	3.04	0.001	3.71
800	54.89	78	44.13	0.21	0.001	2.50
900	57.85	78	42.79	0.10	0.001	3.42
1000	56.29	78	45.01	0.15	0.001	2.05

**Table 3:** Average slice size and Global MSE across the full 10 CTA dataset.

Method	Average Slice Size (kB)	Average MSE
Proposed method with variable bit rate (MVAR)	71.15	3.29
Proposed method with fixed bit rate (MFIX)	61.59	127.16
Lossy JPEG2000 without ROI	73.46	13.86

**Table 4:** Average MOS score for qualitative image quality evaluation for the 10 CTA datasets: (MVAR) proposed method with variable bit rate; (MFIX) proposed method with fixed bit rate; and (JP2K) lossy Jpeg2000 without ROI.

Dataset	Number of Slices	Avg. MOS_MVAR_	Avg. MOS_MFIX_	Avg. MOS_JP2K_
Dataset 1	1038	5	2.7	4
Dataset 2	0985	5	2.8	4.3
Dataset 3	1046	5	2.9	4.2
Dataset 4	1000	5	3.0	4.3
Dataset 5	1029	5	3.0	4.2
Dataset 6	1034	5	2.7	4.1
Dataset 7	0990	5	3.0	4.4
Dataset 8	1009	5	2.6	4.3
Dataset 9	1000	5	2.8	4
Dataset 10	0986	5	3.1	4.1

**Table 5:** Comparative performance evaluation of the four methods for image size reduction evaluation in terms of slice size and compression ratio (CR).

Method	Avg. Slice Size (kB)	Avg. CR
Original file	512	1:1
Lossless JPEG2000	145.95	3.5:1
Lossless JP3D	142.78	3.6:1
Lossless H.264	170.3	3:1
Proposed method with variable bit rate (MVAR)	56.44	9:1

## Data Availability

The raw data supporting the conclusions of this article will be made available by the authors upon request without undue reservation.
